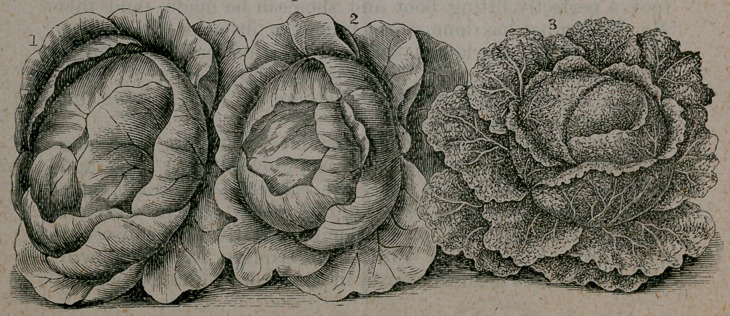# Floral Guide

**Published:** 1875-02

**Authors:** 


VICK'S FLORAL GUIDE, for January, is a beautiful specimen of the printer's art, and is filled with information concerning all manner of seeds and plants, issued quarterly, in Rochester, N. Y., at twenty-five cents a year. It contains 136 pages, 500 engravings, and description of more than 500 of our best flowers and vegetables, with directions for their proper cultivation, among these are cut of several varieties of the familiar and useful Indian corn and cabbage. Mr. Vick's nursery is the largest and most complete in America, from which are promptly furnished every vegetable, seed, cutting, flower, plant and shade tree known in our country. The richness of the information contained in a single quarterly number of the FLORAL GUIDE will surprise the reader.

## Figures and Tables

**Figure f1:**
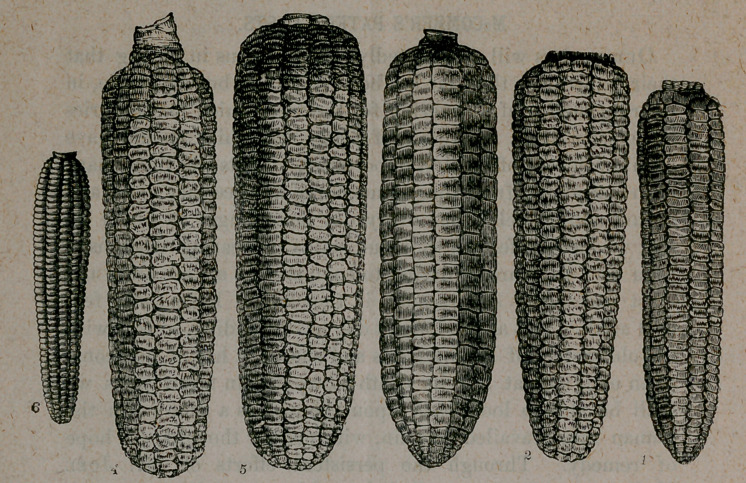


**Figure f2:**